# Suppressing Local Dendrite Hotspots via Current Density Redistribution Using a Superlithiophilic Membrane for Stable Lithium Metal Anode

**DOI:** 10.1002/advs.202206995

**Published:** 2023-02-17

**Authors:** Yifan Hu, Zichuang Li, Zongpeng Wang, Xunlu Wang, Wei Chen, Jiacheng Wang, Wenwu Zhong, Ruguang Ma

**Affiliations:** ^1^ School of Materials Science and Engineering Taizhou University Taizhou 318000 P. R. China; ^2^ State Key Laboratory of High‐Performance Ceramics and Superfine Microstructure Shanghai Institute of Ceramics Chinese Academy of Sciences 1295 Dingxi Road Shanghai 200050 P. R. China; ^3^ Department of Mechanical Materials and Aerospace Engineering Illinois Institute of Technology Chicago Chicago IL 60616 USA; ^4^ School of Materials Science and Engineering Suzhou University of Science and Technology 99 Xuefu Road Suzhou 215009 P. R. China

**Keywords:** alloying, dendrite hotspots, electrospinning, Li metal anodes, reaction, superlithiophilic membranes

## Abstract

Li metal anode is considered as one of the most desirable candidates for next‐generation battery due to its lowest electrochemical potential and high theoretical capacity. However, undesirable dendrite growth severely exacerbates the interfacial stability, thus damaging battery performance and bringing safety concerns. Here, an efficient strategy is proposed to stabilize Li metal anode by digesting dendrites sprout using a 3D flexible superlithiophilic membrane consisting of poly(vinylidene fluoride) (PVDF) and ZnCl_2_ composite nanofibers (PZEM) as a protective layer. Both the experimental studies and theoretical calculations show the origin of superlithiophilicity ascribed to a strong interaction between ZnCl_2_ and PVDF to form the Zn—F bonds. The multifield physics calculation implies effective removal of local dendrite hotspots by PZEM via a more homogeneous Li^+^ flux. The PZEM‐covered Li anode (PZEM@Li) exhibits superior Li deposition/stripping performance in a symmetric cell over 1100 cycles at a high current density of 5 mA cm^−2^. When paired with LiFePO_4_ (LFP), PZEM@Li|LFP full cell remains stable over 1000 cycles at 2 C with a degradation rate of 0.0083% per cycle. This work offers a new route for efficient protection of Li metal anode for practical applications.

## Graphical abstract

Digesting dendrites for stable Li‐metal anode is successfully realized by using a flexible superlithiophilic poly(vinylidene fluoride) and ZnCl_2_ electrospinning membrane as an interfacial protective layer.

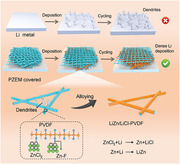

## 1 Introduction

**Figure 1:**
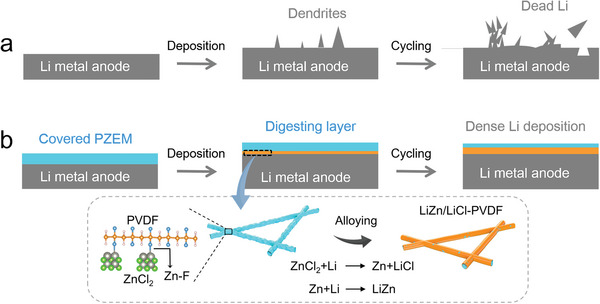
Schematic illustration of Li deposition with and without PZEM. a) A bare Li metal anode enables the continuous growth of Li dendrites in the absence of PZEM. b) PZEM endows Li metal anode with the “digestion” ability for the Li dendrite sprouts, leading to the dense Li deposition without the formation of Li dendrites.

The demands for longer operating time of portable electronic devices and better endurance of electric vehicles have promoted the rapid development of secondary batteries with high energy density.^[^ ^1^ ^]^ Owing to the lowest electrode potential (−3.040 V vs standard hydrogen electrode) and high theoretical capacity (3860 mAh g^−1^), lithium (Li) metal anode shows great potential in the next‐generation lithium‐based batteries.^[^ ^2^ ^]^ Thus, lithium metal batteries (LMBs) have attracted considerable interests of worldwide researchers, heralding the prosperity of Li metal anode as the “Holy Grail” electrode.^[^ ^3^ ^]^ However, the detrimental interface between Li metal and electrolyte leads to the inhomogeneous Li^+^ flux and subsequent dendrite growth during the long‐term cycling as illustrated in **Figure** **1**a.^[^ ^4^ ^]^ These Li dendrites could penetrate through the separator, leading to serious safety issues.^[^ ^5^ ^]^ Moreover, they also easily break away from the electrode, resulting in the formation of “dead Li” and hence the low Coulombic efficiency (CE).^[^ ^6^ ^]^

To stabilize the Li metal anode and suppress the dendrites growth, various ingenious strategies have been proposed by forming in situ solid electrolyte interphase (SEI) layers,^[^ ^7^ ^]^ fabricating 3D hosts,^[^ ^8^, ^9^ ^]^ and introducing external interfacial layers.^[^ ^10^ ^]^ The in situ formation of electrolyte‐derived SEI to protect the Li metal anode was performed by adding functional additives including composite organic lithium salts (e.g., lithium trifluoromethane‐sulfonimide (LiFSI)^[^ ^11^ ^]^ and lithium bis(trifluoromethane)‐sulfonimide (LiTFSI)^[^ ^12^ ^]^), inorganic electrolyte additives (e.g., LiNO_3_^[^ ^5^ ^]^ and CsPF_6_^[^ ^13^ ^]^) and other kinds of substances which can be reduced by Li metal anode (e.g., fluoroethylene carbonate (FEC)^[^ ^14^ ^]^ and vinylene carbonate (VC)^[^ ^15^ ^]^). However, the continuous consumption of sacrificial additives and salts, and poor mechanical strength/flexibility of most SEIs cannot guarantee the long‐term effective protection in the repeated charge–discharge cycles. Many 3D hosts including metal foams (e.g., Cu^[^ ^16^ ^]^ and Ni^[^ ^17^ ^]^) and carbon nanostructures (e.g., carbon nanotube foams^[^ ^18^ ^]^ and graphene films^[^ ^19^ ^]^) have been employed to reduce the local current density of electrode and regulate Li deposition. But their huge specific surface area also leads to severe electrolyte decomposition,^[^ ^20^ ^]^ thus decreasing the CE in the first few cycles. Various interfacial layers as a durable buffer between Li metal anode and electrolyte have also been prepared to solve the dendrite problem, such as flexible organic polymers (e.g., poly(dimethylsiloxane) (PDMS)^[^ ^21^ ^]^ and poly(vinylidene fluoride) (PVDF)^[^ ^22^ ^]^), mechanical inorganic ceramics (e.g., Li_3_N,^[^ ^23^ ^]^ LiPO_3_,^[^ ^24^ ^]^ and Al_2_O_3_^[^ ^25^ ^]^) and their hybrids.^[^ ^26^ ^]^ Although a uniform Li deposition behavior could be observed at a small current density (normally 1 mA cm^−2^), the performance of the assembled LMBs is still far from satisfaction.^[^ ^27^ ^]^ Therefore, it remains challenging to precisely regulate the compositions and microstructures of these ex situ protective layers with strong mechanical strength/flexibility and excellent Li adaptability toward Li metal stabilization.

Herein, we prepare an inch‐scale electrospun membrane consisting of PVDF and ZnCl_2_ composite nanofibers (named as PZEM) that can serve as an interfacial layer to efficiently stabilize the Li metal anode due to increased electrode–electrolyte contact and uniform distribution of Li^+^ flux. ZnCl_2_ anchors in PVDF‐based nanofiber networks and forms a strong chemical bond (Zn—F), which endows PZEM with superlithiophilicity. Once inhomogeneous deposition of Li^+^ (Li dendrites) occurs and proliferates into PZEM, ZnCl_2_ could “digest” the dendrite sprout by LiZn alloying reaction (Figure 1b). Therefore, PZEM with an excellent “digestion” capability can always stay above the deposited lithium to eliminate the dendrites and obtain uniform Li deposition during the cycling (Figure 1b). In stark contrast, a 3D electrospun membrane with single PVDF (named as PEM) cannot robustly mitigate Li epitaxial growth due to a weak affinity with Li metal. Hence, PZEM‐modified Li (PZEM@Li) anode exhibits superior Li deposition/stripping performance in a symmetric cell at either high rate (5 mA cm^−2^) or high capacity (5 mAh cm^−2^). When paired with LFP cathode, the PZEM@Li|LFP full cell achieves a long lifespan over 1000 cycles at 2 C with excellent capacity retention. This PZEM@Li anode was also successfully paired with NCM811 and NCM622 cathodes. We believe that such an interfacial membrane with excellent robustness and stability can offer great prospect for the protection of Li metal anode for the practical application.

## 2 Results and Discussion

**Figure 2:**
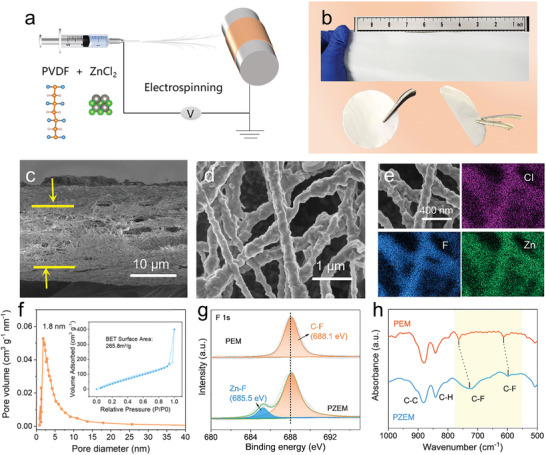
Preparation and characterization of the superlithiophilic PZEM. a) Scheme of a high‐voltage electrospinning apparatus for synthesizing PZEM. b) Digital photos of the flexible PZEM in inch scale. c) Side‐view and d) top‐view SEM images, and e) the corresponding elemental mapping images of Cl, F, and Zn of PZEM. f) Pore size distribution curve and the N_2_ adsorption/desorption isotherms (inset) of PZEM, indicating it is enriched with nanopores and has very high specific surface area of 265.8 m^2^ g^−1^. g) High‐resolution F 1s XPS spectra and h) FTIR spectra of the PZEM and PEM.

PZEM was fabricated through electrospinning as illustrated in **Figure** **2**a. A high voltage was applied between the needle and collector coated with copper foil. And the *N*, *N*‐Dimethylformamide (DMF) solution (10 mL) including ZnCl_2_ (0.3 g) and PVDF (0.8 g) was ejected onto the copper foil to form PZEM. The amount of Zn, Cl, and F in PZEM is estimated to around 13.0, 14.2, and 43.0 wt%, which is in good agreement with the elemental analysis performed by inductively coupled plasma‐mass spectrometry (ICP‐MS) and ion chromatography (IC) (Table S1, Supporting Information). Figure 2b shows a large‐area PZEM with excellent flexibility. Obviously, such a controllable electrospinning route shows a promising prospect to scale up for industrial fabrication. PZEM was punched into wafers for utilization on Li metal. Due to good flexibility, it can accommodate the volumetric expansion of Li deposition to maintain the structural integrity.^[^ ^28^ ^]^ The cross‐sectional scanning electron microscopy (SEM) image of PZEM reveals that PZEM with a uniform thickness of 15 µm possesses a 3D structure composed of nanofibers (Figure 2c). The high‐magnification SEM image shows that these nanofibers have a diameter ranging from 150 to 250 nm. The rough surface could be attributed to the anchored ZnCl_2_ particles, which uniformly distribute within the PVDF‐based polymer networks as confirmed by elemental mapping images (Figure 2e).

In sharp contrast, the as‐electrospun PEM without adding ZnCl_2_ is composed of nanofibers with smooth surface (Figure S1, Supporting Information). The N_2_ adsorption/desorption measurement shows that PZEM exhibits a specific surface area of 265.8 m^2^ g^−1^, and exposes abundant micro/mesopores (1.2–6.2 nm) (Figure 2f). By contrast, PEM without anchored ZnCl_2_ exhibits a neglectable specific surface area of 8.2 m^2^ g^−1^, indicating that there are rare micro/mesopores in PEM.^[^ ^29^ ^]^ It is important to note that abundant porous structure and narrow pore size distribution in PZEM are conducive to homogeneous Li^+^ flux.^[^ ^19^ ^]^ The contact angles for the electrolyte on different samples are illustrated in Figure S2 (Supporting Information). It is clearly seen that after covering PEM and PZEM, the electrodes display much smaller electrolyte contact angles. This demonstrates that PEM and PZEM possess drastically strengthened liquid electrolyte wettability, which could be mainly attributed to the existed C—F bonds with high polarity in the PVDF framework.^[^ ^30^ ^]^ Furthermore, due to the higher surface area, PZEM shows a better electrolyte wettability with a decreased contact angle of 13° compared to 53° of PEM. Such a superior electrolyte wettability for PZEM is also beneficial to the uniform Li^+^ migration.^[^ ^31^ ^]^

X‐ray photoelectron spectroscopy (XPS) was conducted to investigate the chemical states at the surface of PZEM and PEM. As shown in Figure 2g, the deconvoluted peaks in the F 1s spectrum in PZEM are attributed to C—F (688.1 eV) and Zn—F (685.5 eV), while PEM only has the characteristic peak of C—F.^[^ ^32^, ^33^ ^]^ The presence of Zn—F indicates that ZnCl_2_ is incorporated into the PVDF‐based polymer networks by chemical bonding, instead of simply physical adsorption. In the Fourier transform infrared (FTIR) spectra (Figure 2h), an evident change can be detected in the C—F stretching region of PZEM (the yellow area), where the bands at 765 and 615 cm^−1^ redshift slightly and broaden obviously in comparison to those in PEM.^[^ ^34^, ^35^ ^]^ This can also be attributed to the formation of Zn—F. Such a strong interaction between ZnCl_2_ and PVDF helps endow high homogeneity and stability with PZEM.

**Figure 3:**
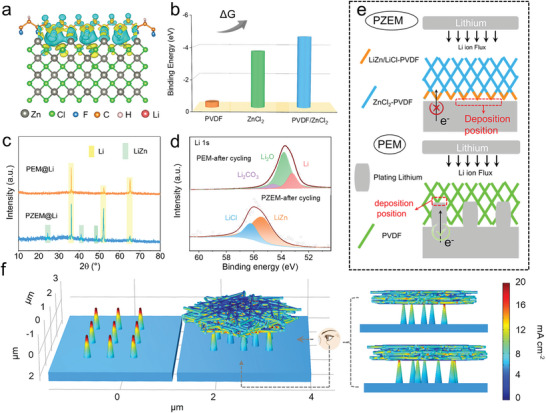
Elucidating PZEM superlithiophilicity by combining theoretical calculations with experimental studies. a) Differential charge density distribution of PVDF binding with ZnCl_2_ (PVDF/ZnCl_2_). b) Binding energy of Li atoms with PVDF, ZnCl_2_, and PVDF/ZnCl_2_. c) XRD patterns of PEM@Li and PZEM@Li electrodes after 10 cycles in symmetric cells under a current density of 1 mA cm^−2^ and capacity of 1 mAh cm^−2^. d) High‐resolution Li 1s XPS spectra of the cycled PEM and PZEM, peeled off from Li foil after 10 cycles in symmetric cells. e) Schematic illustration of the Li deposition on PZEM@Li (up panel) and PEM@Li electrode (down panel). f) COMSOL simulation of the electric field distribution of Li dendrites with and without PZEM covered on the Li anode.

To explore the effect of PZEM on the surface of Li metal, density functional theory (DFT) calculations were conducted. The corresponding differential charge density distribution of PZEM shows remarkable charge transfer from F to Zn (**Figure** **3**a). And the bond length of C—F in PVDF increases from 1.375 to 1.458 Å after incorporating ZnCl_2_ into the membrane. These results further confirm the substantial interaction between F and Zn. Based on optimized geometrical structures (Figure S3, Supporting Information), the lithiophilicity of PZEM and PEM was studied as presented in Figure 3b. Impressively, the binding energy of Li atoms anchoring at different substrates increases in the order: PVDF (−0.35 eV) < ZnCl_2_ (−3.69 eV) < ZnCl_2_‐PVDF (−4.64 eV). This manifests that PZEM is more lithiophilic than PEM due to the enhanced binding energy of Li and ZnCl_2_.^[^ ^36^ ^]^ The existence of Zn—F can not only enable high homogeneity and stability, but also induce superlithiophilicity of PZEM.

Because PZEM and PEM are highly insulative (Figure S4, Supporting Information), Li migration would take place through such membranes when they are used as a protective layer on Li metal. As a result, the electrodeposition of Li happens underneath PZEM/PEM, which is proved by the cross‐sectional SEM images of PZEM@Cu and PEM@Cu anodes after Li deposition of 4 mAh cm^−2^ (Figure S5, Supporting Information). Due to the high reactivity of Li metal, the dendritic electrodeposits could appear on the surface of the anode during the electrodeposition (Figure S5a, Supporting Information).^[^ ^37^ ^]^ If the protective layer is covered, Li dendrites would grow up to pierce into it, and the lithiophilic site inside could serve as a dendrite absorbent by chemical reaction. Owing to the porous 3D structure, there are plenty of contact sites in PZEM for lithium nucleation. With superlithiophilicity, it could entirely eliminate the Li dendrites inside the membrane, so a dense Li deposition layer is obtained between PZEM and Cu (Figure S5b, Supporting Information). In contrast, due to low lithiophilicity of PEM, Li dendrites growth cannot completely be interrupted in PEM, and subsequent Li deposition is embedded inside PEM nanofiber network to cover the formed dendrites. Hence, there is no obvious interface between deposited Li and PEM (Figure S5c, Supporting Information).

In order to distinguish the growth mechanism of Li dendrite in PZEM and PEM, PZEM@Li, and PEM@Li electrodes were studied after 10 cycles in symmetric cells under the current density of 1 mA cm^−2^ and capacity of 1 mAh cm^−2^. The X‐ray diffraction (XRD) patterns clearly show characteristic peaks of Li metal and LiZn alloy in PZEM@Li (Figure 3c), whereas only Li metal exists in PEM@Li. The emergence of LiZn alloy means that the removal of dendrites is realized by alloying reaction between ZnCl_2_ and Li. This is well consistent with the DFT calculations showing that the superlithiophilicity in PZEM mainly originates from ZnCl_2_. XPS analysis was performed on the cycled PZEM/PEM which is peeled off from Li foil. As shown in Figure 3d, the Li 1s spectrum of PEM exhibits a peak at 53.1 eV assigned to Li embedded inside the membrane.^[^ ^38^ ^]^ Some LiOH/Li_2_CO_3_ (54.8 eV) and Li_2_O (53.7 eV) signals also present, which might arise from the reaction of pristine Li metal with electrolyte.^[^ ^39^ ^]^ The presence of LiCl (56.3 eV) and LiZn (55.5 eV) is evidenced in the Li 1s spectrum of PZEM.^[^ ^40^ ^]^ Interestingly, LiCl is not visible in the XRD pattern of the PZEM@Li electrode due to its low scattering factor or amorphous nature. Based on these analyses, it can be concluded that the elimination of Li dendrites in PZEM is based on an in situ alloying reaction between Li and ZnCl_2_. SEM image of the cycled PZEM indicates that the morphology of the nanofibers changes after reaction with the Li dendritic (Figure S6a, Supporting Information). The originally wrinkled surface of nanofibers becomes smooth, like being coated by a layer, which possibly results from alloying reaction upon Li adsorbed onto nanofibers.^[^ ^41^ ^]^ As for PEM, SEM image reveals that Li deposition is obvious inside the nanofibers network after cycling (Figure S6b, Supporting Information), which is consistent with the above analysis. Large volume deposition is also conducted for PZEM, as shown in Figure S7 (Supporting Information). Li metal is further deposited inside the host, without dendrite formation or aggregation phenomenon. The host is robust enough to show excellent accommodation capacity for Li deposition, indicating enough mechanical strength.

To clearly demonstrate the difference, Li deposition behaviors on PZEM@Li and PEM@Li are described in Figure 3e. After the initial cycle, a layer containing LiCl, LiZn, and PVDF forms at the bottom of PZEM. As the LiZn alloy with good electrical conductivity is wrapped by highly insulating LiCl and PVDF, the whole layer still keeps being electrically insulative.^[^ ^40^ ^]^ So, Li deposition could only arise at the surface of Li foil to lift up PZEM. This indicates a strong stability of interface between PZEM and Li metal, which effectively inhibits the growth of Li dendrites upon repeating Li deposition/stripping process. In sharp contrast, the chemically inert and electronically insulating PVDF nanofiber network in PEM only acts as a scaffold to accommodate the deposition of Li. Li^+^ could travel the shortest pathway through the scaffold to gain electrons, and Li metal could progressively fill the overhead space to grow toward the separator.^[^ ^42^ ^]^ Once Li metal appears at the top of PEM, the following deposition will not be guided and confined, and Li dendrites will grow in an uncontrolled fashion.^[^ ^43^ ^]^ So, PEM with a limited thickness (15 µm) cannot effectively suppress the Li dendrites, especially under the high‐capacity Li deposition. Thus, it can be seen that different from PEM, PZEM could stay above the deposited Li at all times to tackle the dendrite growth.

Moreover, by using COMSOL Multiphysics, we simulated the current density distribution to explore how PZEM affects the dendrite growth under it. As shown in Figure 3f, when there is no protective layer covered, a dramatical increase in current density can be observed in the tips of the protuberant dendrites, which could induce the continuous growth of lithium dendrites.^[^ ^44^ ^]^ Under the same conditions, the introduction of PZEM can eliminate the self‐amplification behavior in the tip of the protuberant dendrites by redistributing the current density, which leads to a more homogeneous incoming Li^+^ flux.^[^ ^45^ ^]^ By removing the local hotspot of dendrites and accommodating more dendrites due to superlithiophilicity, PZEM provides double insurances to guarantee even and dense Li deposition, which is quite different from the protective layers reported previously.

The symmetric cells with bare Li and PZEM@Li electrodes were disassembled after 10 cycles under the current density of 1 mA cm^−2^ and capacity of 1 mAh cm^−2^ to analyze the formed SEI on Li foil. Note that PZEM@Li electrode was washed by DMF to expose the formed SEI. The corresponding element concentrations detected by XPS are shown in Table S2 (Supporting Information). It is noted that the PZEM modification enables a significant increase of F content from 27.92 to 43.14 at% and a decrease of C content from 16.64 to 6.49 at%. The F 1s and Li 1s spectra of the modified SEI shows that the enrichment of F exists in the form of LiF (Figure S8, Supporting Information).^[^ ^46^ ^]^And compared with the SEI formed at the surface of bare Li, a higher fraction of LiF in the modified SEI by PZEM is observed, which originates from reaction between fluorine groups in PVDF and Li foil. Among different components of SEI, LiF is deemed to be steady due to its excellent electronic insulation, high mechanical modulus, and proper diffusion barrier.^[^ ^47^ ^]^ The enrichment of LiF in SEI is beneficial to decreasing byproducts derived from the electrolyte decomposition,^[^ ^39^ ^]^ which is evidenced by the dilution of C content and weakened intensity of C 1s spectrum (Figure S9, Supporting Information). From the above analysis, we conclude that the PZEM modification could induce a LiF‐rich stable SEI layer to suppress side reactions between Li metal and electrolyte, which is vitally important for long‐term stability of Li metal interface.^[^ ^48^ ^]^

**Figure 4:**
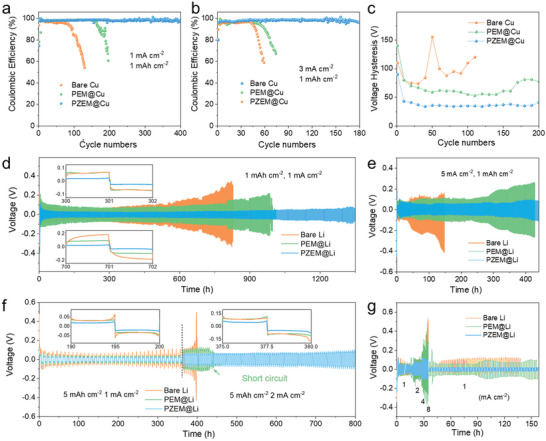
Electrochemical performances of Li|Cu cells. a,b) CE of Li|Cu cells using bare Cu, PEM@Cu, and PZEM@Cu electrodes at a) 1 mA cm^−2^ and b) 3 mA cm^−2^ with a plating capacity of 1 mAh cm^−2^. c) Comparison of voltage hysteresis of the Li plating/stripping at 1 mA cm^−2^ for bare Cu, PEM@Cu, and PZEM@Cu electrodes. d–f) Voltage profiles of the symmetric cells with bare Li, PEM@Li, and PZEM@Li electrodes at d) 1 mA cm^−2^, e) 5 mA cm^−2^ for 1 mAh cm^−2^, and f) different current densities from 1 to 2 mA cm^−2^ for 5 mAh cm^−2^. g) Rate capability under various current densities from 1 to 8 mA cm^−2^ using bare Li, PEM@Li, and PZEM@Li electrodes. The results indicate the superlithiophilic PZEM significantly improves the CE and cycling stability of Li metal.

Coulombic efficiency (CE) known as an important index sensitive to dendrites was tested by Li/Cu cells paired with bare Cu, PEM@Cu, and PZEM@Cu electrodes.^[^ ^49^ ^]^ As shown in **Figure** **4**a, at a current density of 1 mA cm^−2^ with a capacity of 1 mAh cm^−2^, the Li/Cu cells with different anodes were cycled. Due to the dendrite growth and the formation of “dead” Li, the bare Cu electrode shows a rapid drop in CE only after 80 cycles. The modified PEM@Cu electrode exhibits an improved CE of 97.2% after 150 cycles, but an abrupt drop appears afterward. This is related to the characteristics of PEM. Once the Li deposition occurs at the surface of PEM, the structural stability of PEM@Cu would deteriorate sharply. The PZEM@Cu can sustain 400 cycles with an average CE of 98.3% for 400 cycles, which is much more stable than the PEM@Cu and bare Cu electrodes. As discussed above, PZEM with superlithiophilicity could solve the dendrite problem through homogenizing the Li^+^ distribution, thus obtaining a stable electrode/electrolyte interface. As a result, the removal of “dead” Li contributes to high CE during the long‐term cycling.^[^ ^50^ ^]^ At a raised density of 3 mA cm^−2^, the CE of the PZEM electrode still reaches 97.7% for more than 180 cycles, which is much superior to other two electrodes (Figure 4b). Results summarized in Table S3 (Supporting Information) shows a fascinating performance of PZEM@Cu, which is competitive to those previously reported anodes. Li nucleation overpotentials are compared at 1 mA cm^−2^ for different Cu electrodes (Figure S10, Supporting Information). Note that the nucleation overpotentials for the modified electrodes become smaller than bare Cu electrode, which could originate from the better electrode–electrolyte contact owing to the abundant fluorine groups in PVDF.^[^ ^31^ ^]^ Voltage profiles of three electrodes at 1 mA cm^−2^ with a cycling capacity of 1 mAh cm^−2^ are shown in Figure S11 (Supporting Information). Voltage hysteresis defined as the sum of the overpotential for Li deposition and Li stripping is compared in Figure 4c. Impressively, PZEM@Cu owns a minimal voltage hysteresis, which maintains at 45 mV over 200 cycles. Compared with PEM@Cu, smaller voltage hysteresis of PZEM@Cu during initial cycles could be attributed to its higher specific surface area, implying positive effects of open pores on reducing the overall charge‐transfer resistance (*R*_ct_).^[^ ^29^ ^]^ In the subsequent cycles, by eliminating the dendrites and inhibiting its growth, PZEM@Cu shows high stability and induces uniform Li deposition to keep small overpotential, coinciding with the stable high CE.

The electrochemical performances were further evaluated in symmetric cells. Figure 4d presents a comparison of the electrochemical Li deposition/stripping behavior of PZEM@Li, PEM@Li, and bare Li electrodes at 1 mA cm^−2^ with a capacity of 1 mAh cm^−2^. Due to the elimination of Li dendrites and improved stability of the electrode/electrolyte interface, PZEM@Li exhibits better cycling stability over ≈1360 h with lower potential hysteresis (inset of Figure 4d). In comparison, PEM@Li and bare Li electrodes suffer from a drastic voltage increase and short circuit within much shorter time. Even at a raised current density of 5 mA cm^−2^, PZEM@Li still demonstrates a remarkable cyclic stability over 1100 cycles (Figure 4e), which outperforms a majority of recently reported Li anodes modified by interfacial protective layers (Table S4, Supporting Information). The rate performance in Figure 4g indicates that PZEM@Li exhibits a much lower overpotential and flat voltage plateau at different current densities. However, bare Li and PEM@Li exhibit substantial voltage fluctuation during the Li deposition/stripping process, demonstrating their increased polarization.^[^ ^51^ ^]^ As shown in Figure 4f, when the Li deposition/stripping is increased to larger capacity, PZEM@Li anode still shows enough stability. During the beginning of cycling at the current density of 1 mA cm^−2^ for 5 mAh cm^−2^, PZEM@Li owns a lower hysteresis than bare Li and PEM@Li electrodes (inset of Figure 4f). After 36 cycles, the current density increases to 2 mA cm^−2^, PZEM@Li maintains almost unchanged plating overpotentials of 46 mV for subsequent 88 cycles. But for bare Li and PEM@Li, the hysteresis rises abruptly and the cell fails within remarkably short time. Such wonderful high‐rate and high‐capacity performance of PZEM@Li electrodes verifies the robustness and stability to cope with the dendrites problem. Electrochemical impedance spectroscopy (EIS) measurement was conducted to further prove the interfacial stability in PZEM modified symmetric cell (Figure S12, Supporting Information). It can be seen that bare Li and PEM@Li electrode show high and ever‐increasing *R*_ct_, which could be attributed to the accumulation of electronically inactive “dead” Li evoked by growing dendrites.^[^ ^45^ ^]^ Interestingly the *R*_ct_ of PEM@Li is higher than bare Li with a low current density, indicating a higher polarization, which also explains the voltage profiles of the symmetric cells in Figure 4. For PZEM@Li, the *R*_ct_ is much lower and remains almost constant during the cycling, because not only abundant pore structure in PZEM provides fast Li^+^ transport channel, but also the elimination of Li dendrites gets rid of “dead” Li.^[^ ^52^ ^]^ This indicates a stable interface on Li metal surface modified by PZEM, which is consistent with the lower polarization observed in Figure 3d–g.

**Figure 5:**
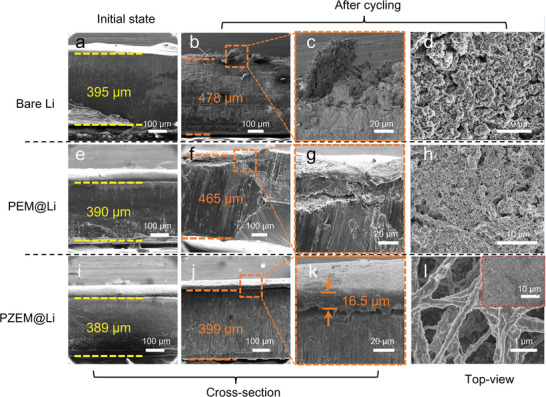
The morphology changes of bare Li, PEM@Li, and PZEM@Li before (initial state) and after cycling. Cross‐section SEM images of a–c) bare Li, e–g) PEM@Li, and i–k) PZEM@Li electrode before and after cycling. Top‐view SEM images of d) bare Li, h) PEM@Li, and l) PZEM@Li electrode after cycling. The inset in l) is the SEM image of the same piece of the cycled PZEM@Li anode with exposed deposited Li at the top surface. In this case, PZEM was removed by washing using DMF.

The thickness variation and surface morphology of bare Li, PEM@Li, and PZEM@Li electrodes in symmetric cell during the cycling were characterized by SEM. As shown in **Figure** **5**a,b, cross‐section SEM images show the thickness fluctuation of bare Li is up to 83 µm (21% volume expansion) after 100 cycles at 1 mA cm^−2^, which could be caused by dendrite growth and formation of “dead” Li.^[^ ^53^ ^]^ Local enlarged image (Figure 5c) and top‐view image (Figure 5d) further confirm the presence of mossy‐like dendrites and pulverization phenomenon. This loosely connected structure with deleterious volume change also appeared for PEM@Li after cycling. As shown in Figure 5e,f, the thickness fluctuation is up to 75 µm (19% volume expansion). Local enlarged image in Figure 5g indicates that Li is deposited through PEM. From the top‐view image (Figure 5h), mossy‐like dendrites with a few fibers spread over the surface. PEM fails to induce dense Li deposition, because the inhibition effect of PEM on vertical dendrite growth is only limited by its thickness. Once the Li deposition occurs at the surface of PEM, it would be unable to guide and confine the subsequent Li deposition. Different from above two electrodes, PZEM@Li shows very tiny volume change (2% volume expansion) after cycling (Figure 5i,j). Instead of a scaffold, PZEM stays above the Li metal serving as an artificial protective layer to facilitate homogeneous Li deposition (Figure 5k). And the top‐view image (Figure 5l) demonstrates that nanofiber network in the membrane is well preserved after cycling. The thickness of PZEM (16.5 µm) slightly increases compared with uncycled sample (≈15 µm), which could be attributed to the introduction of alloying products inside the membrane by reacting with Li dendrites. After PZEM@Li was washed with DMF to remove PZEM, dense Li metal microstructure without obvious dendrites was observed (inset of Figure 5l). These findings once again verify the important role of PZEM in effectively eliminating Li dendrites and improving uniformity of Li deposition. It is noted that the thickness of PZEM should affect the performance of Li deposition. By changing electrospinning time, membranes that are half or twice as thick as PZEM are produced. As shown in Figure S13 (Supporting Information), at a current density of 2 mA cm^−2^ with a capacity of 2 mAh cm^−2^, the Li/Cu cells with different thickness of PZEM were cycled. When the membrane is too thick, the anode would show a slow ion transfer kinetics, which increase the production of dead lithium.^[^ ^23^ ^]^ When the membrane is too thin, the capacity of accommodating dendrites could be reduced.^[^ ^14^ ^]^ So the even and dense Li deposition cannot be guaranteed during long cycling. It is obvious that PZEM with a uniform thickness of 15 µm shows best performance when used as an interfacial protective layer for Li metal.

**Figure 6:**
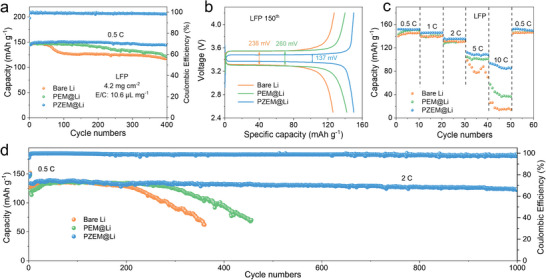
Cycling stability and rate capability of full cells with different anodes (bare Li, PEM@Li, and PZEM@Li) and cathodes (LFP). a) Cycling stability and b) voltage profiles of PZEM@Li|LFP, PEM@Li|LFP, and Li|LFP at 0.5 C with LFP loading of 4.2 mg cm^−2^. c) Rate performance of PZEM@Li|LFP, PEM@Li|LFP, and Li|LFP at various rates from 0.5 to 10 C. d) Long‐cycling performances of PZEM@Li|LFP, PEM@Li|LFP, and Li|LFP at 2 C.

The electrochemical performance was further tested in full cells by coupling three anodes (bare Li, PEM@Li, and PZEM@Li) with LiFePO_4_ (LFP). As shown in **Figure** **6**a, after cycling at 0.5 C, PZEM@Li|LFP delivers a very stable capacity without obvious fluctuation, and retains 144.1 mAh g^−1^ after 400 cycles, 99.6% of the original capacity. By contrast, bare Li|LFP and PEM@Li|LFP show capacity decay only after 50 or 160 cycles, and retains 77.3% or 80.2% of initial capacity after 400 cycles. Such different cycling performance further corroborates that the PZEM modification leads to a more stable electrode–electrolyte interface with the elimination of dendrites, while PEM@Li and bare Li suffer from severe dendrite growth and interfacial side reactions.^[^ ^54^ ^]^ The galvanostatic voltage profiles (Figure 6b), exhibit reduced voltage hysteresis in PZEM modified LFP cells at 0.5 C, again suggesting faster charge‐transfer behavior and better stability at the electrode interface. The enhanced rate performance is also observed in PZEM@Li|LFP with a reversible capacity of 151.9, 145.8, 136.3, 112.2, and 93.7 mAh g^−1^ at 0.5, 1, 2, 5, and 10 C, respectively, which are much higher than those of bare Li|LFP and PEM@Li|LFP (Figure 6c). Remarkably, PZEM@Li|LFP sustains up to 1000 cycles with a decay rate of 0.0083% per cycle at 2 C (Figure 6d). Likewise, full cells by coupling LiNi_0.8_Co_0.1_Mn_0.1_O_2_ (NCM811) and LiNi_0.6_Co_0.2_Mn_0.2_O_2_ (NCM622) with three anodes were also tested. As shown in Figure S14a (Supporting Information), after cycling at 0.2 C for the first two cycles activation and 0.5 C for the following cycles, PZEM@Li|NCM811 delivers a very stable capacity without obvious fluctuation, and retains 168.2 mAh g^−1^ after 150 cycles, 94.3% of the original capacity. By contrast, bare Li|NCM811 and PEM@Li|NCM811 exhibit sharply decreased capacity and obtain low capacity of 102.7 and 121.4 mAh g^−1^ after 150 cycles, with only 60.4% and 67.5% capacity retention. The galvanostatic voltage profiles show reduced voltage hysteresis in PZEM modified NCM811 cells (Figure S14b, Supporting Information). PZEM@Li|NCM811 also delivers an excellent rate performance and reaches 93.7 mAh g^−1^ even at 10 C, superior to bare Li|LFP and PEM@Li|LFP which only deliver the specific capacity of 14.3 and 36.8 mAh g^−1^, respectively (Figure S14c, Supporting Information). NCM622 cells under the commercially relevant conditions (low negative/positive electrode capacity (N/P) ratio and lean electrolyte content) were also tested to further gauge the compatibility and practicability of PZEM in full batteries.^[^ ^55^ ^]^ As shown in Figure S14d (Supporting Information), PZEM@Li|LFP achieves a relatively stable capacity retention (86.5%) after 70 cycles, while Li|NCM622 and PEM@Li|NCM622 exhibit a continuous capacity fading at 0.3 C. These distinct performances of PZEM@Li anode suggest its great potential in the practical applications.

## 3 Conclusions

In summary, a 3D protective layer consisting of composite nanofibers with ZnCl_2_ and PVDF has been successfully fabricated by electrospinning to accomplish homogeneous deposition of Li and highly efficient Li metal anode. The resultant PZEM has improved electrolyte wettability and appropriate porous structure to facilitate uniform Li^+^ flux and reduce interface resistance. ZnCl_2_ chemically anchors on the PVDF framework and induces superlithiophilicity of PZEM, so as to “digest” Li dendrites by alloying reaction. Furthermore, PZEM with excellent robustness and stability is observed to stay above the deposited Li during cycling, which affords shielding effect to remove the local hotspot of dendrites under it. Consequently, there are no obvious dendrites at the surface of the PZEM‐modified anode. And such an anode retains a high average CE (98.3%) in a Li|Cu cell for more than 400 cycles, and exhibits superior Li deposition/stripping performance in a symmetric cell for more than 1100 cycles at a high current density of 5 mA cm^−2^. When paired with LFP and NCM811 cathodes, the full cell with PZEM@Li anode shows high‐capacity retention and excellent rate performances. Even under harsh condition (low N/P, lean electrolyte, and high mass loading), PZEM@Li|NCM622 full cell shows improved cyclability compared with its counterparts. This work provides new guidance to effectively stabilize the Li/electrolyte interface, which will push forward the application of LMBs.

## Conflict of Interest

The authors declare no conflict of interest.

## Supporting information

Supporting Information

## Data Availability

The data that support the findings of this study are available on request from the corresponding author. The data are not publicly available due to privacy or ethical restrictions.
