# Supplementary material for: Suppressing Local Dendrite Hotspots via Current Density Redistribution Using a Superlithiophilic Membrane for Stable Lithium Metal Anode
Source: Adv Sci (Weinh). 2023 Feb 17;10(12):2206995. doi: 10.1002/advs.202206995 (PMC10131806; doi:10.1002/advs.202206995)
Supplement: Supplementary file 1 — Supporting Information [file ADVS-10-2206995-s001.pdf]

ADVANCED SCIENCE
Open Access

Supporting Information

for *Adv. Sci.*, DOI 10.1002/advs.202206995

Suppressing Local Dendrite Hotspots via Current Density Redistribution Using a Superlithiophilic Membrane for Stable Lithium Metal Anode

Yifan Hu, Zichuang Li, Zongpeng Wang, Xunlu Wang, Wei Chen, Jiacheng Wang, Wenwu Zhong* and Ruguang Ma**

Supplementary Materials

Suppressing Local Dendrite Hotspots *via* Current Density Redistribution using A Superlithiophilic Membrane for Stable Lithium Metal Anode

Yifan Hu,[1,2] Zichuang Li,[2] Zongpeng Wang,[1] Xunlu Wang,[2] Wei Chen,[3] Jiacheng Wang,[1, 2*] Wenwu Zhong,[1*] and Ruguang Ma [2, 4*]

1. School of Materials Science and Engineering, Taizhou University, Taizhou 318000, China. Email: zhongww@tzu.edu.cn
2. State Key Laboratory of High-Performance Ceramics and Superfine Microstructure, Shanghai Institute of Ceramics, Chinese Academy of Sciences, 1295 Dingxi Road, Shanghai 200050, China. E-mail: maruguang@mail.sic.ac.cn; jiacheng.wang@mail.sic.ac.cn
3. Department of Mechanical Materials and Aerospace Engineering, Illinois Institute of Technology Chicago, IL 60616, USA
4. School of Materials Science and Engineering, Suzhou University of Science and Technology, 99 Xuefu Road, Suzhou 215009, China

Experimental Section

Preparation of Electrospun Membrane: 0.8 g PVDF (average Mw = 534000 g mol^{-1}) was dissolved in 10 mL N,N-Dimethylformamide (DMF) and stirred at 45 °C for 6 h to achieve complete solvation. Afterward 0.3 g $ZnCl_2$ was added and stirred at 75 °C for 12 h until a homogeneous precursor solution was obtained. 0.4 mL h^{-1} solution feed rate, 22–25 kV applied voltage, and 15 cm distance between the stainless steel spinneret and collector were set as the key electrospinning parameters. The temperature and relative humidity were maintained at 45 °C and 35%, respectively. The resulting electrospun membrane (named PZEM) was peeled off from collector and hot pressed at 75 °C and 10 MPa to reach a uniform membrane thickness of around 15 µm. PEM was prepared without addition of $ZnCl_2$.

Materials characterizations: The structure and morphology of the obtained samples were characterized by Powder X-ray diffraction (XRD) (D8 ADVANCE, Cu Kα radiation), scanning electron microscopy (SEM) (FEI Magellan 400). The chemical composition of the products was analyzed by X-ray photoelectron spectroscopy (XPS) measurements (ESCALAB) using Al Kα (hν = 1486.6 eV) radiation. The nitrogen sorption measurements were performed by adsorption of liquid nitrogen at 77 K by a McMurray Tristar ii 3020 analyzer. The surface areas and pore sizes were calculated using the Brunauer–Emmett–Teller (BET) and Barrett–Joyner–Halenda (BJH) methods, respectively. Fourier transform infrared spectroscopy (Bruker VERTEX 70 FTIR spectrometer) were used to characterize the composition of the obtained samples. Inductively coupled plasma mass spectrometry (ICP-MS) measurements were carried out on a Agilent 7700x. Ion chromatography measurements were carried out on a Dionex ICS-6000 HPIC.

Electrochemical measurement: Electrochemical performance was tested by CR2016-type coin cells on battery testing system (Land battery tester, Wuhan, China). The coin cells were assembled in the argon-filled glove box with O_2 and H_2O content below 0.1 ppm and Celgard 2400 was used as the separator. The Li|Cu half cells were assembled by using Cu and Li foils as the counter electrodes. To standardize, 50 μL of 1.0 M lithium bis(trifluoromethanesulfonyl) imide (LiTFSI) in 1,3-dioxolane/1,2-dimethoxyethane (DOL/DME, v:v=1:1) solution with 1 wt% $LiNO_3$ additive was used in the Li|Cu cells and symmetric Li|Li cells. Electrochemical impedance spectrometry (EIS) measurements were carried out at the frequency4 range of 100 KHz to 0.01 Hz using Chenhua electrochemical workstation. The $LiFePO_4$ (LFP), $LiNi_{0.8}Co_{0.1}Mn_{0.1}O_2$ (NCM811) and $LiNi_{0.6}Co_{0.2}Mn_{0.2}O_2$ (NCM622) cathodes were made by blending active materials, carbon black and polyvinylidene fluoride binder (PVDF) with a weight ratio of 8: 1: 1 to form slurry. The areal mass loading of LFP, NCM811 and NCM622 on the electrodes was 4.2, 8.4 and 21.6 mg cm^{-2}, respectively. The electrolyte added in Li|LFP, Li|NCM811 and Li|NCM622 full cells was 50 μL of 1.0 M $LiPF_6$ in ethylene carbonate/diethyl carbonate solution (EC/DEC, v:v=1:1). For the Li|NCM622 full cells cycled under the harsh condition, 50 μL of 1.0 M $LiPF_6$ in EC/DEC solution was used as the electrolyte, and ultrathin Li metal foils (50 μm) were used as the anodes. The galvanostatic charge-discharge processes were conducted on Land battery cyclers (Wuhan, China).

COMSOL Simulation and DFT Calculation: COMSOL multiphysics software with lithium battery module was used to simulate current density distributions of half-cells with or without PZEM. According to the experimental characterization results, the nanofibers were stacked irregularly and the diameter was set as 250 nm. The length of dendrite was set as 2 μm. To make the simulation process as close as possible to the actual situation, the current density in bulk electrolyte was set to 10 mA cm^{-2} and the resulting ionic current distribution was taken at steady-state.[1] The electrolyte conductivity was set to 1.805×10^{-3} S cm^{-1}, and the Li^+ diffusion coefficient was 10^{-8} m S^{-2}. The initial Li^+ concentration was set to 1 M.

DFT calculations were performed using the Vienna ab initio simulation package (VASP). The generalized gradient approximation method with the Perdew–Burke–Ernzerhof (PBE) exchange–correlation functional was used to manage the electron exchange and correlation energy.[2] And the kinetic energy cut-off was set to be 450 eV. A mesh of 2 * 2 * 1 was used for the k-point sampling obtained from the Gamma center. Meanwhile, the model is 11.9 * 15.8 * 34.2 $Å^3$, and the thickness of the vacuum layer is 30 Å. Geometries were optimized until the energy and the force were less than 1.0×10^{-4} eV per atom and -0.0257 eV $Å^{-1}$, respectively. The interaction strength between Li and the substrates (such as $ZnCl_2$(001), PVDF, and PVDF bonding with $ZnCl_2$) was described by the adsorption energy, E_{ads}, which is defined as

$$E_{ads} = E_{Li-substrate} - E_{substrate} - E_{Li}$$

where $E_{Li\text{-}substrate}$, $E_{substrate}$, and E_{Li} are the energies of Li adsorbed substrate, the isolated substrate, and the single Li atom, respectively.

The differential charge density distribution (ρ_{diff}) in PVDF bonding with $ZnCl_2$ is calculated as follows

$$\rho_{diff} = \rho_{PVDF-ZnCl_2} - \rho_{ZnCl_2} - \rho_{PVDF}$$

where the visualization was processed using the VESTA package.

Supplementary Figures

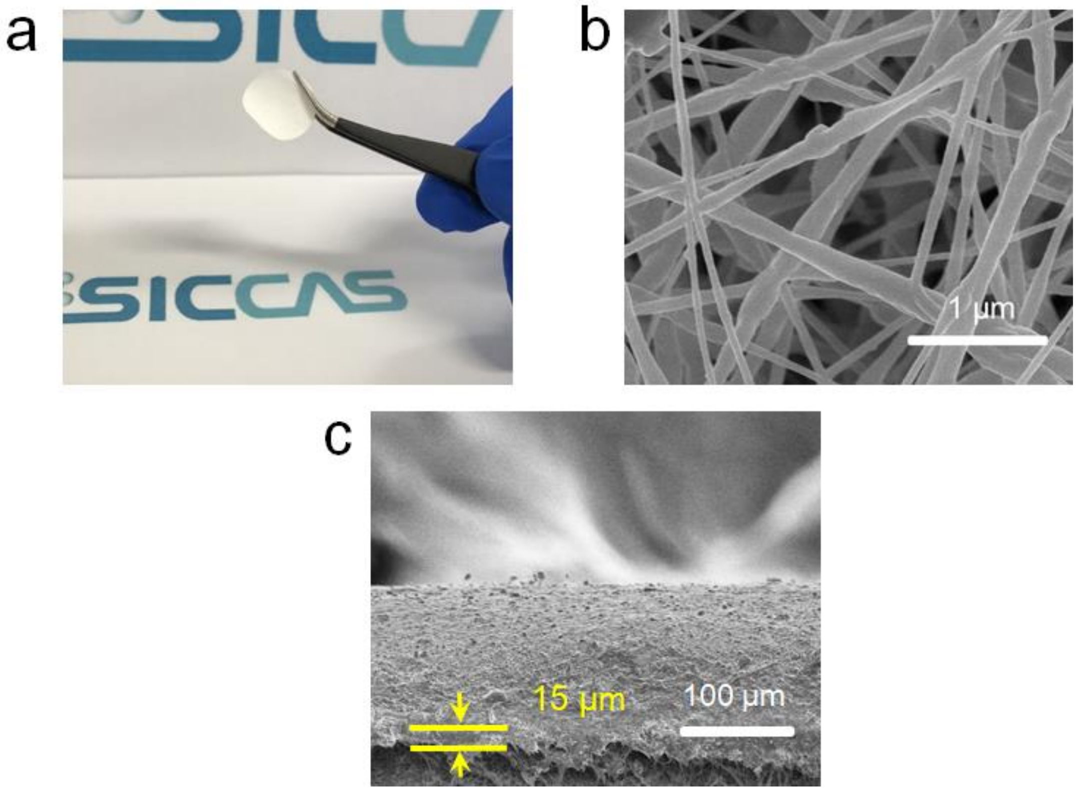

Supplementary Fig.1. (a) Digital photos of PEM. (b) Top-view and (c) side-view SEM images of PEM.

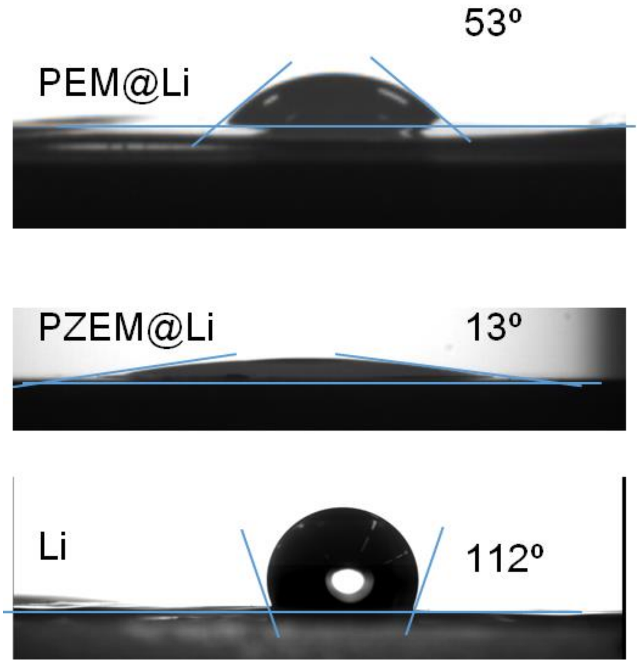

Supplementary Fig.2. Contact angles of electrolyte toward different anodes.

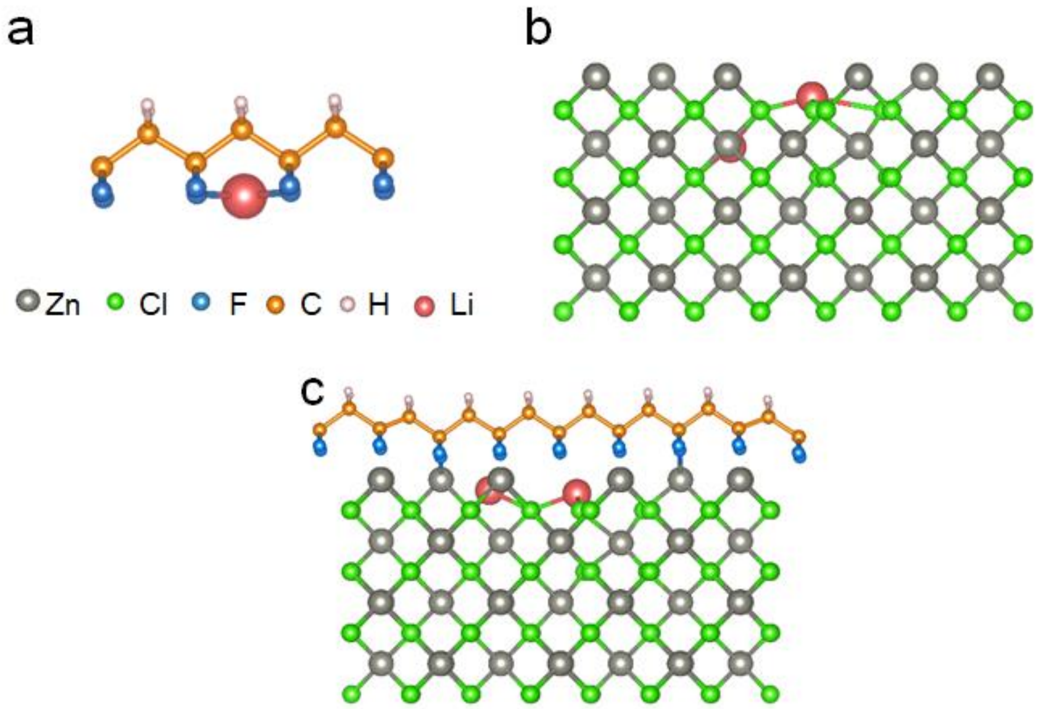

Supplementary Fig.3. Optimized geometrical structures of Li atoms adsorbed on (a) PVDF, (b) $ZnCl_2$, and (c) $ZnCl_2$-PVDF.

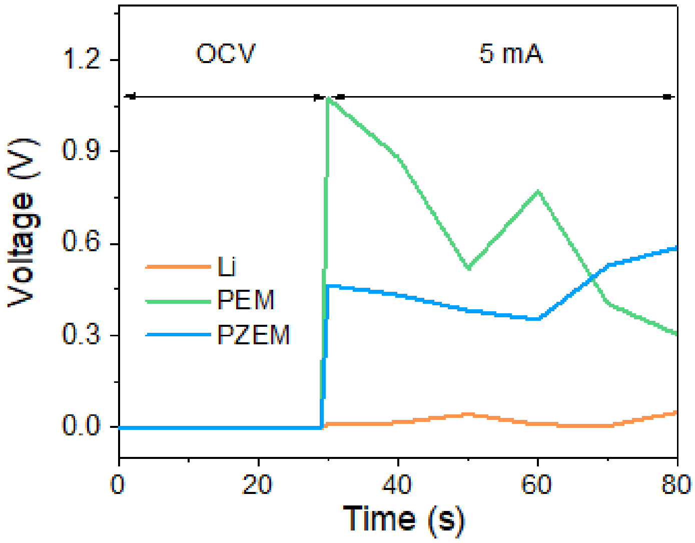

Supplementary Fig.4. Voltage response of bare Li (orange curve), PEM/Li/PEM (green curve) and PZEM/Li/PZEM (blue curve) electrodes at an applied current of 5 mA.

Note: Electrical resistivity of modified lithium electrodes was calculated as following: (*L*-thickness of the composite protection film; *I*–applied current; *S*-area of the contact between stainless steel and the alloy; *U*–average voltage increase):

$$\rho = \frac{\mathrm{U \cdot S}}{\mathrm{I \cdot L}}$$

The total thickness of the PEM/PZEM membrane is 15/15 μm. The calculated values of electronic resistivity for PEM and PZEM are $9.9*10^4$ and $6.9*10^4$ Ω cm, respectively.

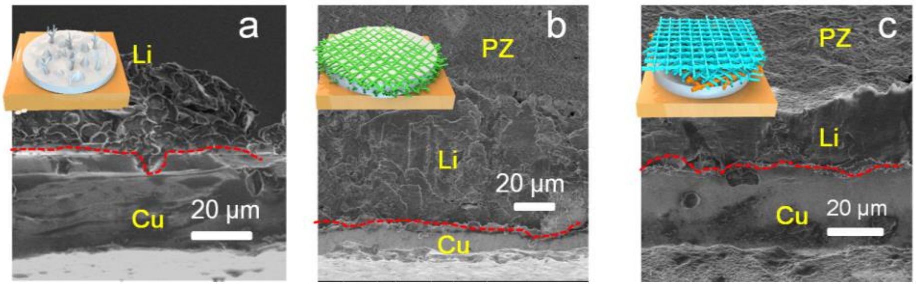

Supplementary Fig.5. Cross-sectional SEM images and the corresponding schematic drawing of (a) bare Cu, (b) PEM@Cu and (c) PZEM@Cu anodes after 4 mAh cm^{-2} Li deposition.

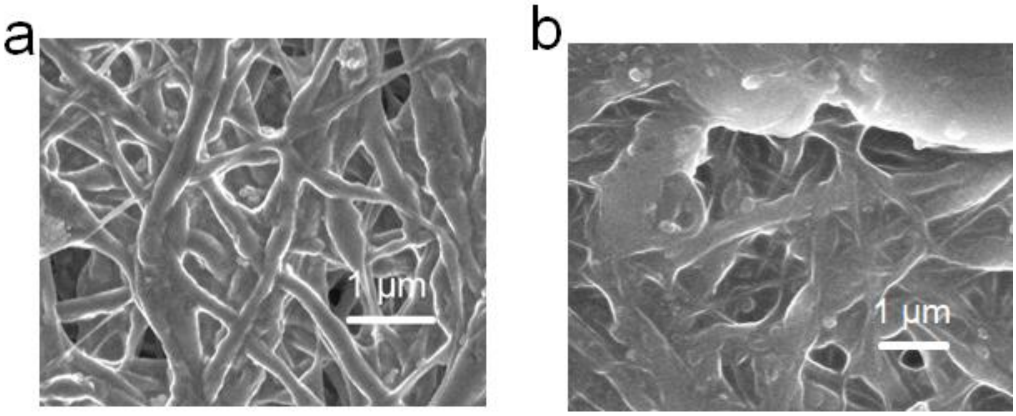

Supplementary Fig.6. SEM images of (a) PZEM and (b) PEM after 10 cycles in symmetric cells at the current density of 1 mA cm^{-2} and capacity of 1 mAh cm^{-2}.

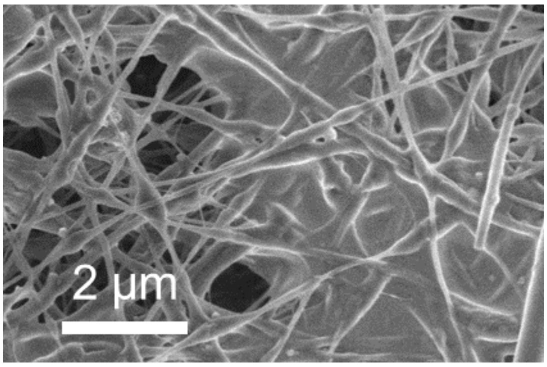

Supplementary Fig.7. SEM images of (a) PZEM after Li deposition of 4 mAh cm^{-2}.

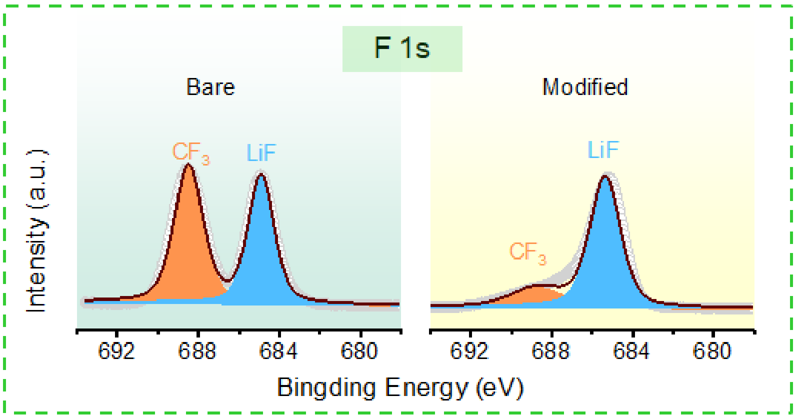

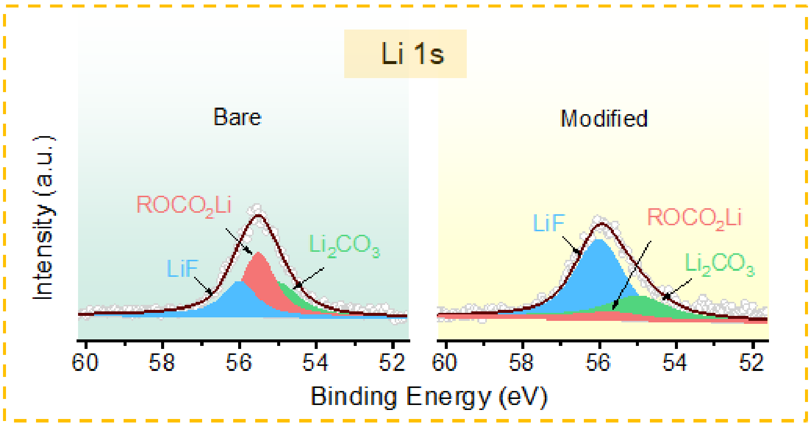

Supplementary Fig.8. F 1s and Li 1s XPS spectra of SEI formed with and without PZEM modification.

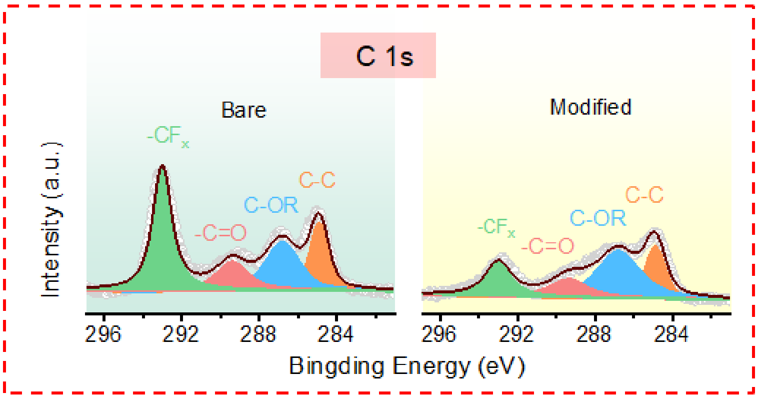

Supplementary Fig.9. C 1s XPS spectra of SEI formed with and without PZEM.

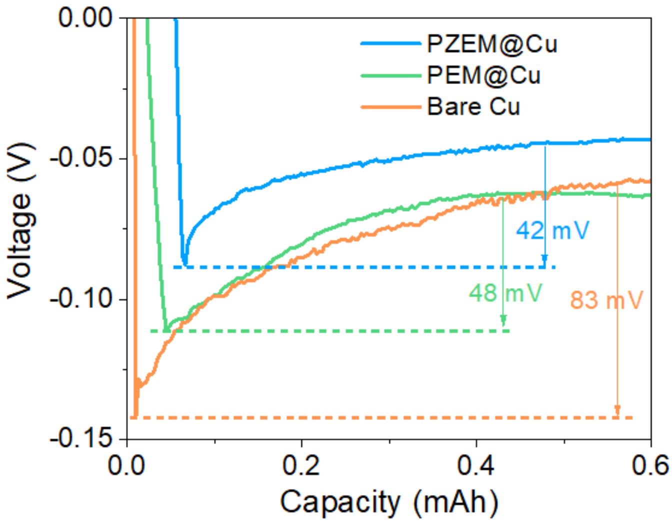

Supplementary Fig.10. Nucleation overpotentials of Li deposition on bare Cu, PEM@Cu, and PZEM@Cu electrodes.

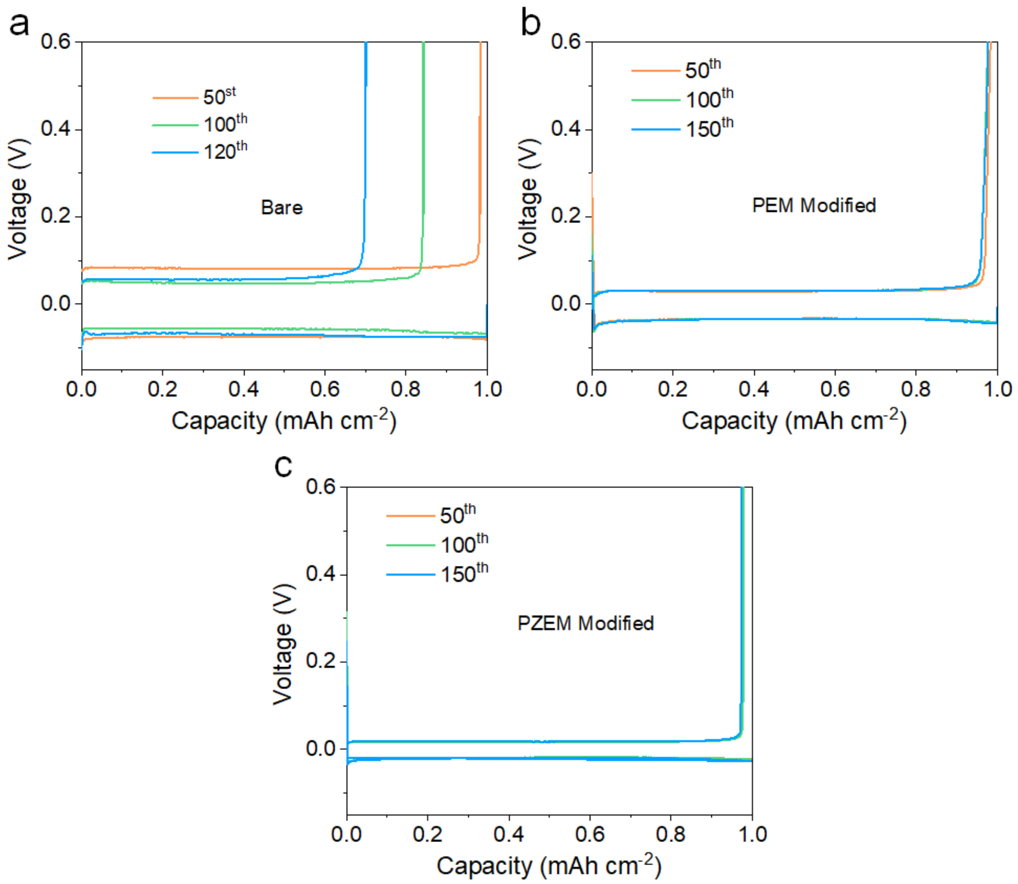

Supplementary Fig.11. Voltage profiles of (a) bare Cu, (b) PEM@Cu, and (c) PZEM@Cu electrodes at different cycles.

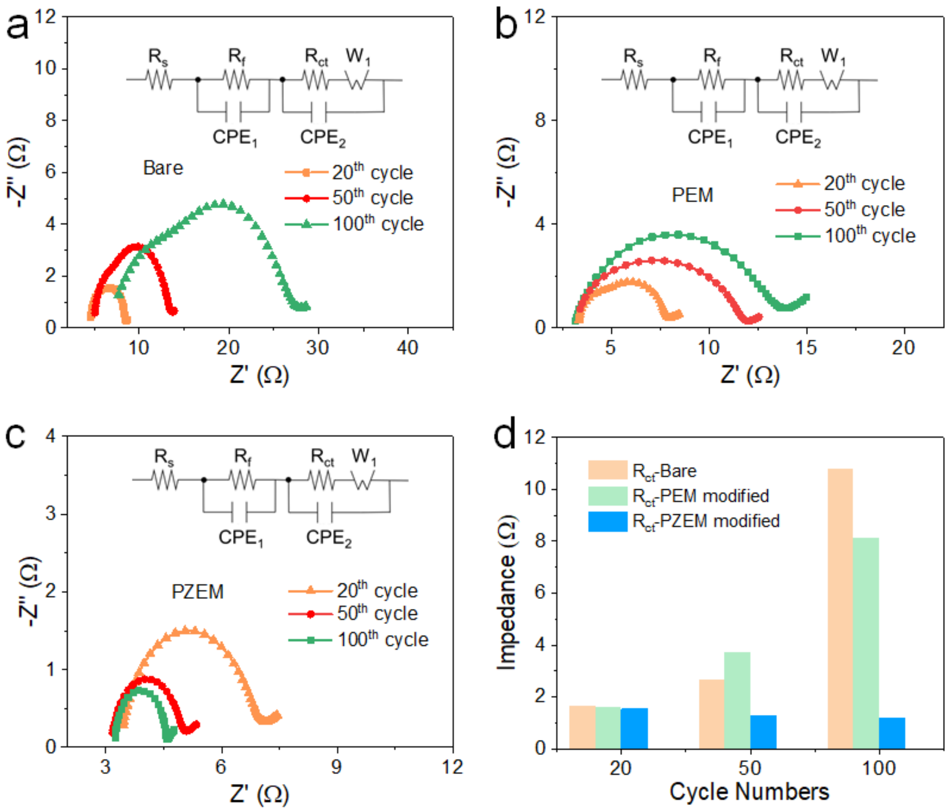

Supplementary Fig.12. EIS curves and the corresponding equivalent circuits of (a) bare Li, (b) PEM@Li and (c) PZEM@Li in symmetric cells at 1 mA cm^{-2} and 1 mAh cm^{-2}. (d) Comparison of R_{ct} values depending on different cycle number.

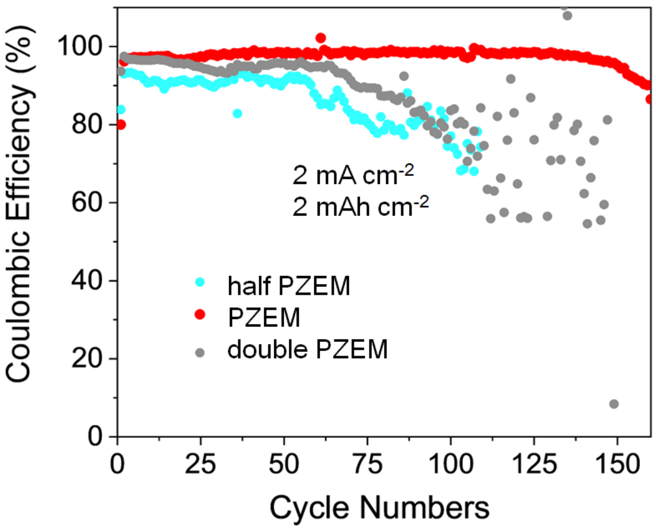

Supplementary Fig.13. CE of Li|Cu cells using PZEM@Cu electrodes at 2 mA cm^{-2} and with a plating capacity of 2 mAh cm^{-2}. By changing electrospinning time, membranes that are half or twice as thick as PZEM are produced.

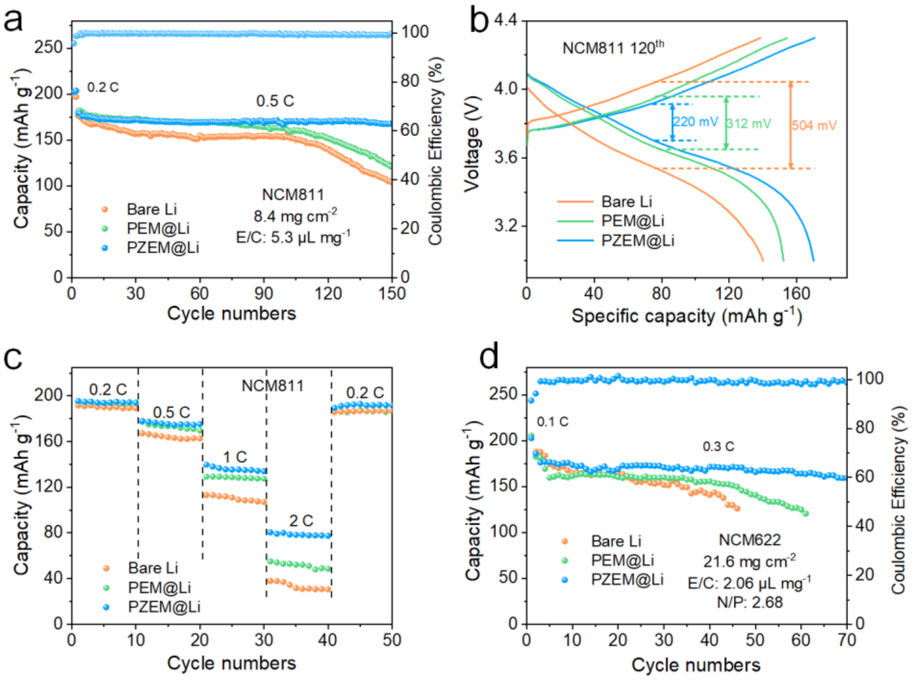

Supplementary Fig.14. (a) Cycling stability and (b) voltage profiles of PZEM@Li|NCM811, PEM@Li|NCM811 and Li|NCM811 at 0.5 C with NCM811 loading of 4 mg cm^{-2}. (c) Rate performance of PZEM@Li|NCM811, PEM@Li|NCM811 and Li|NCM811 at various rates from 0.2 to 2 C. (d) Cycling stability of PZEM@Li|NCM622, PEM@Li|NCM622 and Li|NCM622 at 0.5 C with NCM622 high loading of 21.6 mg cm^{-2}.

Supplementary Table 1. Zn, Cl and F content determined by ICP-MS and IC for PZEM.

	ICP (wt.%)	IC (wt.%)
Zn	12.5	-
Cl	-	13.7
F	-	42.9

Supplementary Table 2. Chemical composition for SEI formed with and without PZEM modification.

	XPS composition (at.%)					
	**Li**	**C**	**O**	**F**	**S**	**N**
Bare	30.13	16.64	16.48	27.92	5.33	3.49
Modified	36.69	6.49	8.28	43.14	2.73	2.67

Supplementary Table 3. The comparison of the electrochemical performances of the Li|Cu cells using different interfacial protective layers reported in the literatures. Lifespan indicates the cycles during which the Coulomb efficiency remains stable.

**Interfacial protective layers**	**Current density (mA cm^{-2})**	**Capacities (mAh g^{-1})**	**Lifespan (cycles)**	**Reference**
PZEM	1	1	400	This work
β-PVDF	1	0.5	300	ACS Energy Lett. **6**, 4416 (2021)
F-PPTA@PP	1	1	200	Adv. Energy Mater **12**, 2202206 (2022)
PDDA–TFSI	1	1	120	Adv. Mater. **33**, 2007428 (2021)
CuF_2	1	1	300	J. Energy Chem **37**, 29 (2019)
PZEM	1	3	180	This work
SF-PVA	1	3	150	Adv. Funct. Mater. **31**, 2100537 (2021)
CuQDs	1	2	150	Adv. Mater. **32**, 2004379 (2020)
g-C_3N_4	1	1	140	*Adv. Energy Mater.* **10**, 2002647 (2020)

Supplementary Table 4. The comparison of the electrochemical performances of the symmetric cells using different interfacial protective layers reported in the literatures.

**Interfacial protective layers**	**Current density (mA cm^{-2})**	**Capacities (mAh g^{-1})**	**Lifespan (cycles)**	**Reference**
PZEM	5	1	1100	This work
Hybrid polyuria	5	1	150	Adv. Energy Mater. **10**, 2001139 (2020)
$[LiNBH]_n$ layer	3	1	1050	Adv. Funct. Mater. **30**, 2002414 (2020)
UCLN	5	1	750	Adv. Energy Mater **11**, 2003496 (2021)
LiF(one-pot solution)	5	1	150	Energy Storage Mater. **16**, 85 (2019)
Dual-layered film	5	1	100	Adv. Mater. **30**, 1707629 (2018)
Hydrophobic GF-LiF layer	5	1	125	Nat. Commun. **10**, 900 (2019)
Li_3Sb alloy layer	5	1	370	Chem. Mater. **31**, 7565 (2019)
ZrO_2	5	1	225	Angew. Chem. Int. Ed. **58**, 15797 (2019)
Alucone	5	1	750	Small Methods, **2**, 1700417 (2018)
PVDF-HFP/LiF	5	1	250	Adv. Funct. Mater. **28**, 1705838 (2018)

References

S[1] S. Li, Q. Liu, J. Zhou, T. Pan, L. Gao, W. Zhang, L. Fan, Y. Lu. *Adv. Funct. Mater.* **2019**, *29*, 1808847.

S[2] Q. Wang, C. Yang, J. Yang, K. Wu, C. Hu, J. Lu, W. Liu, X. Sun, J. Qiu, H. Zhou. *Adv. Mater.* **2019**, *31*, 1903248.
